# A Combined Experimental and Mathematical Approach for Molecular-based Optimization of Irinotecan Circadian Delivery

**DOI:** 10.1371/journal.pcbi.1002143

**Published:** 2011-09-08

**Authors:** Annabelle Ballesta, Sandrine Dulong, Chadi Abbara, Boris Cohen, Alper Okyar, Jean Clairambault, Francis Levi

**Affiliations:** 1 INRIA Rocquencourt, BANG project team, Le Chesnay Cedex, France; 2 INSERM, U776 “Rythmes biologiques et cancers”, Hôpital Paul Brousse,Villejuif, France; 3 Université Paris-Sud, UMR-SO776, Orsay, France; 4 Assistance Publique - Hôpitaux de Paris, Unité de Chronothérapie, Département d'oncologie médicale, Hôpital Paul Brousse, Villejuif, France; 5 Istanbul University Faculty of Pharmacy, Department of Pharmacology, Istanbul, Turkey; Max Planck Institute for Informatics, Germany

## Abstract

Circadian timing largely modifies efficacy and toxicity of many anticancer drugs. Recent findings suggest that optimal circadian delivery patterns depend on the patient genetic background. We present here a combined experimental and mathematical approach for the design of chronomodulated administration schedules tailored to the patient molecular profile. As a proof of concept we optimized exposure of Caco-2 colon cancer cells to irinotecan (CPT11), a cytotoxic drug approved for the treatment of colorectal cancer. CPT11 was bioactivated into SN38 and its efflux was mediated by ATP-Binding-Cassette (ABC) transporters in Caco-2 cells. After cell synchronization with a serum shock defining Circadian Time (CT) 0, circadian rhythms with a period of 26 h 50 (SD 63 min) were observed in the mRNA expression of clock genes *REV-ERBα*, *PER2*, *BMAL1*, the drug target topoisomerase 1 (*TOP1*), the activation enzyme carboxylesterase 2 (*CES2*), the deactivation enzyme UDP-glucuronosyltransferase 1, polypeptide A1 (*UGT1A1*), and efflux transporters *ABCB1*, *ABCC1*, *ABCC2* and *ABCG2*. DNA-bound TOP1 protein amount in presence of CPT11, a marker of the drug PD, also displayed circadian variations. A mathematical model of CPT11 molecular pharmacokinetics-pharmacodynamics (PK-PD) was designed and fitted to experimental data. It predicted that CPT11 bioactivation was the main determinant of CPT11 PD circadian rhythm. We then adopted the therapeutics strategy of maximizing efficacy in non-synchronized cells, considered as cancer cells, under a constraint of maximum toxicity in synchronized cells, representing healthy ones. We considered exposure schemes in the form of an initial concentration of CPT11 given at a particular CT, over a duration ranging from 1 to 27 h. For any dose of CPT11, optimal exposure durations varied from 3h40 to 7h10. Optimal schemes started between CT2h10 and CT2h30, a time interval corresponding to 1h30 to 1h50 before the nadir of CPT11 bioactivation rhythm in healthy cells.

## Author Summary

Treatment timing within the 24-h timescale, that is, circadian (circa, about; dies, day) timing, can change by several fold the tolerability and antitumor efficacy of anticancer agents both in experimental models and in cancer patients. Chronotherapeutics aims at improving the tolerability and/or the efficacy of medications through the administration of treatments according to biological rhythms. Recent findings highlight the need of individualizing circadian delivery schedules according to the patient genetic background. In order to address this issue, we propose a combined experimental and mathematical approach in which molecular mathematical models are fitted to experimental measurements of critical biological variables in the studied experimental model or patient. Optimization procedures are then applied to the calibrated mathematical model for the design of theoretically optimal circadian delivery patterns. As a first proof of concept we focused on the anticancer drug irinotecan. A mathematical model of the drug molecular PK-PD was built and fitted to experimental data in Caco-2 colon cancer cells. Numerical algorithms were then applied to theoretically optimize the chronomodulated exposure of Caco-2 cells to irinotecan.

## Introduction

Circadian timing largely modifies efficacy and toxicity of many anticancer drugs. Chronomodulated administration schemes for patients have been designed based on chronotoxicity results obtained in mice and subsequently validated in clinical trials in which all patients have received the same regimen. However recent findings highlight the need of personalizing circadian delivery according to the patient gender and genetic background [1], [2]. The systems biology approach presented here aims at designing optimal chronotherapeutics schedules using mathematical models fitted to the patient molecular profile. We propose here an *in vitro* proof of concept which focuses on irinotecan (CPT11), a cytotoxic drug approved for the treatment of colorectal cancer [3]. CPT11 efficacy and toxicity display circadian rhythms in mice [4], [5] and in patients [6], [7]. Its circadian administration is here optimized in cell culture using a combined experimental and mathematical approach.

Most biological functions in mammals such as rest-activity, body temperature or hormonal secretions, display rhythms of period between 20 and 28 h called circadian rhythms. Circadian changes are coordinated by the suprachiasmatic nuclei (SCN), an endogenous pacemaker located in the hypothalamus. SCN functions display an intrinsic genetically-determined period which is entrained and calibrated at precisely 24 h by environmental synchronizers such as the alternation of days and nights, socio-professional routines and meal timing [8]. This central pacemaker controls through rhythmic physiological signals the molecular circadian clock present in each nucleated cell. The cellular molecular clock is constituted of interconnected regulatory loops involving about 15 clock genes such as *CLOCK*, *PER*, *BMAL*, or *REV-ERB* 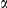. Those genes display circadian rhythms in their expression and generate in turn circadian oscillations of various gene and protein amounts. [9]. In particular, many enzymes involved in drug metabolism, cell cycle, DNA repair or apoptosis display circadian variations and induce rhythms in the toxicity and efficacy of many anticancer drugs. The circadian organization is often disrupted in tumor tissues. This temporal difference between normal and cancer cells is exploited in cancer chronotherapeutics by targeting the circadian time of minimum toxicity in healthy cells [8].

*In vitro* chronotherapeutics studies derive their rationale from the fact that each nucleated cell is endowed with a molecular circadian clock. Nevertheless, in the absence of external synchronizer, the millions of cells contained in a Petri dish oscillate neither with the same phase nor with the same period [10], [11]. Synchronization with a serum shock (exposure to a large amount of nutrients [12]), drugs [13] or temperature cycles [14] resets the cellular clocks which then oscillate in synchrony with a circadian period. Of note, the serum shock may activate transcription factors which induce a transient overshoot in the expression of some genes during the first periods.

As CPT11 is highly toxic for the colon mucosa and efficient against colorectal adenocarcinomas, the human colon cancer cell line Caco-2 was chosen for this *in vitro* study. Caco-2 cells constitute a well-established cellular model for investigating both colon physiology and colon cancer susceptibility to drugs. Furthermore, they do express clock genes [15].

**Figure 1:**
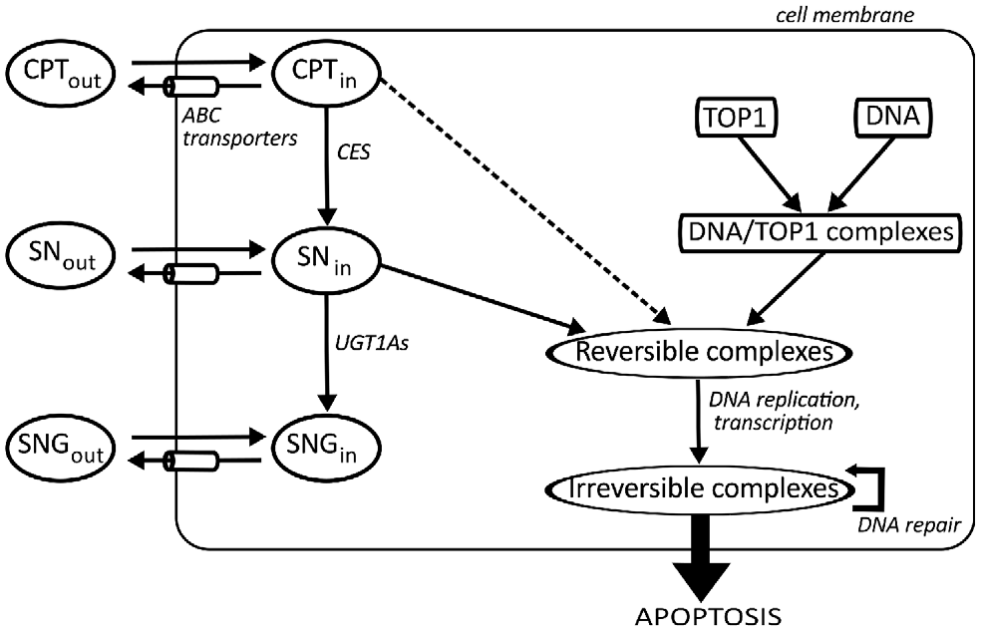
CPT11 PK-PD molecular pathways considered in Caco-2 cells. CPT11 in the extracellular medium (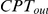) diffuses passively through the cell membrane and reaches the intracellular compartment (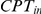). It is then bioactivated into SN38 (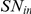) through CESs. 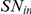 is deactivated into SN38G (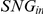) through UGT1As. 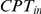, 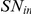 and 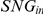 are expelled outside of the cell by ABC transporters (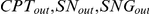). 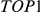 is an enzyme which relaxes supercoiled DNA by creating transient *DNA/TOP1 complexes*. 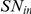 traps them into SN38/DNA/TOP1 *reversible complexes* which becomes irreversible after collision with replication or transcription mechanisms, thus triggering DNA repair and possibly apoptosis.

Concerning CPT11 PK, the drug is bioactivated by CESs into SN38 which is 100 to 1000-fold more cytotoxic (Figure 1, [16], [17]). SN38 is deactivated into SN38G by glucuronidation through mainly UGT1A1 [18] and other UGT1As [19]. CPT11 cellular uptake is passive in intestinal cells whereas that of SN38 occurs passively only at low pH when the carboxylate form is predominant. The uptake of SN38 lactone form is possibly mediated by active mechanisms [20], [21]. CPT11 and its metabolites are actively expelled outside of the cells by transporters of the ABC super-family. CPT11 is preferentially transported by ABCB1, ABCC1 and ABCC2; SN38 by ABCG2, ABCC1 and ABCC2; and SN38G by ABCC2 and ABCG2 [22], [23].

CPT11 is an inhibitor of TOP1, an enzyme present in all nucleated cells (Figure 1, [24]). Its function is to relax DNA which may be supercoiled by several processes including replication and transcription. TOP1 binds to DNA and cuts one strand which is thus able to rotate around the molecule. Then TOP1 dissociates from DNA allowing the reconnection of the broken strand. CPT11 and its active metabolite SN38 prevent TOP1 religation by creating DNA/TOP1/drug complexes which can spontaneously dissociate but have a longer lifetime than DNA/TOP1 complexes. Collisions between those ternary reversible complexes and replication or transcription mechanisms convert them into irreversible covalent DNA damage which triggers DNA repair and possibly leads to cell cycle arrest and apoptosis [24], [25]. The amount of TOP1 complexes on the DNA has been experimentally correlated to CPT11 cytotoxicity both *in vitro* and in patients [26], [27].

Several genes and proteins involved in CPT11 PK-PD display circadian rhythms in mice including the drug target *top1*, the activation enzymes *ces1* and *ces2*, the deactivation enzyme *ugt1a1* and the efflux transporters *abcb1a*, *abcb1b* and *abcc2* [2], [28]–[30].

We implemented here a combined experimental and mathematical approach for optimizing CPT11 circadian delivery according to the critical molecular determinants of its chronotoxicity.

## Results

### CPT11 PK-PD in non-synchronized cells

**Figure 2:**
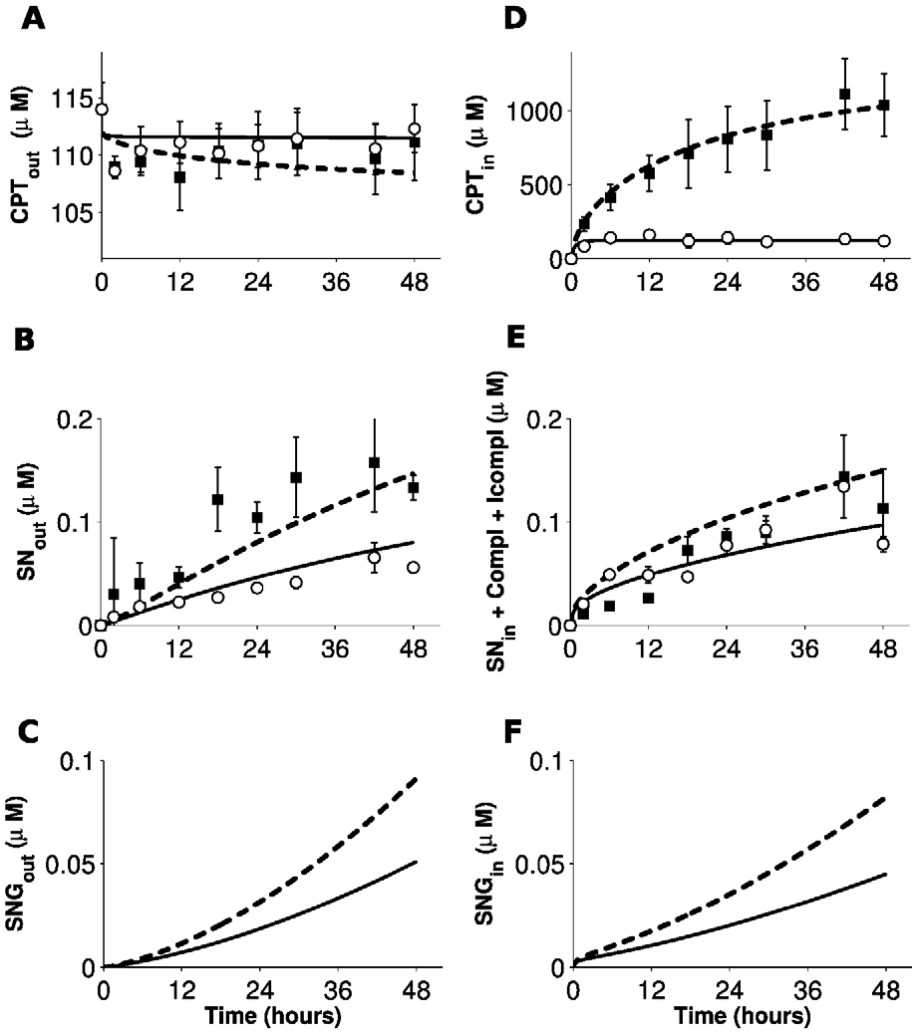
Time evolution of CPT11, SN38 and SN38G extra- and intracellular concentrations during CPT11 exposure. Drug concentrations in absence (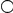) or presence (▪) of verapamil are averages of four data points obtained from two independent experiments (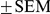). Solid and dashed curves are the best fits of the PK-PD mathematical model in absence or presence of verapamil respectively. **A**,**B**,**C**: extracellular concentrations of CPT11, SN38 and SN38G; **D**, **E**, **F**: intracellular concentrations of CPT11, SN38 and SN38G (sum of free and DNA-bound molecules).

CPT11 accumulated into non-synchronized Caco-2 cells (Figure 2). Approximately 0.1% of its total amount was bioactivated into SN38. Verapamil drastically increased the intracellular accumulation of CPT11 and slightly decreased its extracellular concentration. This confirmed the influence on CPT11 efflux of ABCB1, ABCC1 and ABCC2, three transporters inhibited by verapamil. The increase in CPT11 intracellular concentration in presence of verapamil resulted in increased SN38 production. Nevertheless SN38 intracellular concentration remained similar whereas SN38 extracellular concentration increased in presence of the inhibitor which therefore did not alter SN38 efflux.

### Circadian control of CPT11 PK-PD in synchronized cells

**Figure 3:**
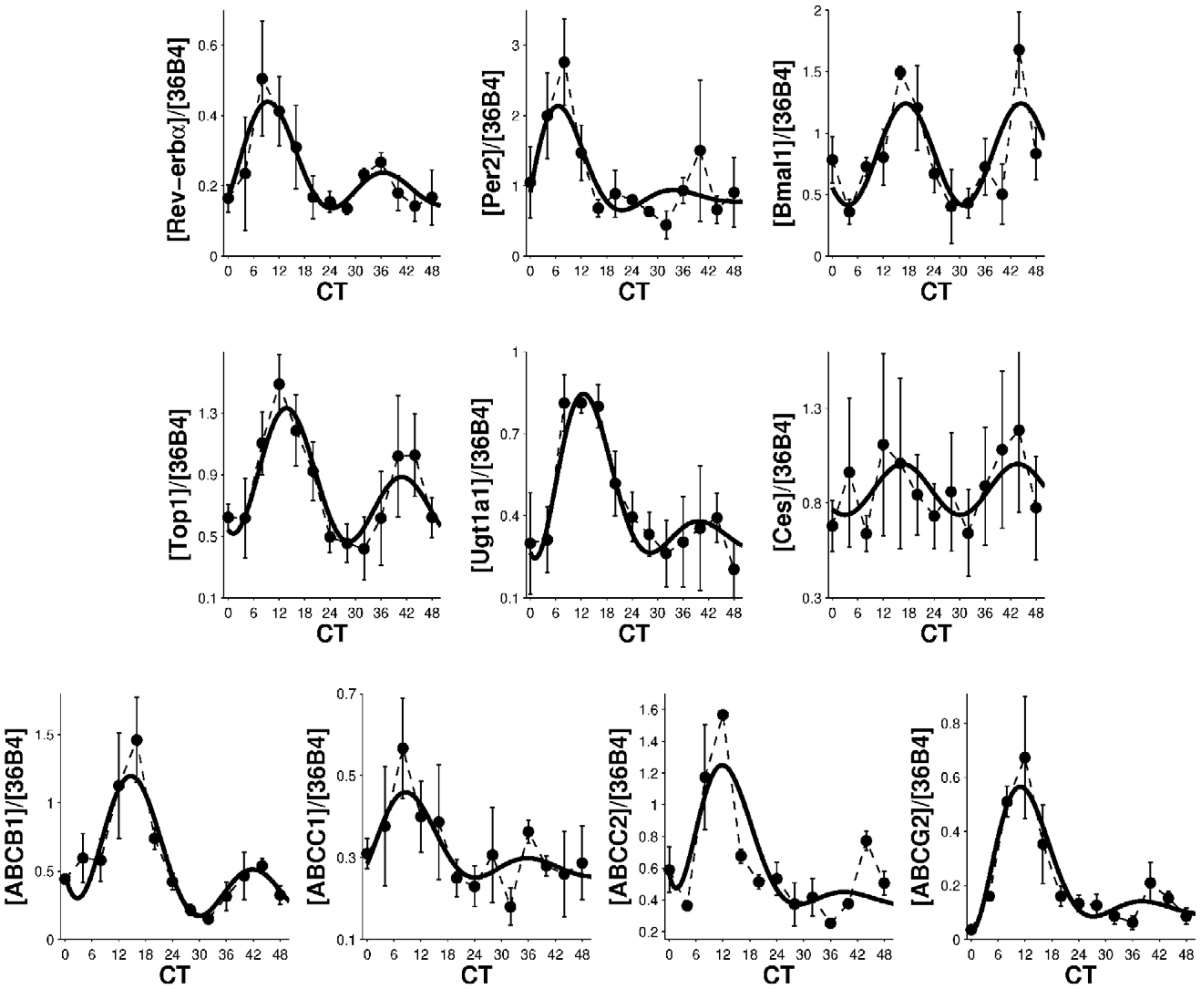
Gene expression circadian rhythm in synchronized Caco-2 cells. mRNA level of three clock genes (*REV-ERB* 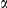, *PER2*, *BMAL1*) and seven genes involved in CPT11 PK-PD (*TOP1*, *UGT1A1*, *CES2*, *ABCB1*, *ABCC1*, *ABCC2*, *ABCG2*) displayed circadian variations. Experimental results are averages of four independent experiments for *REV-ERB* 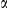, *TOP1*, *UGT1A1*, and *CES2*; and three independent experiments for *PER2*, *BMAL1*, *ABCB1*, *ABCC1*, *ABCC2*, and *ABCG2* (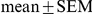). The solid curve is the best fit of equation 12 (see Table 1 for parameter values).

Three clock genes (*REV-ERB* 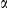, *PER2*, *BMAL1*) and seven pharmacological genes (*TOP1*, *UGT1A1*, *CES2*, *ABCB1*, *ABCC1*, *ABCC2*, *ABCG2*) displayed circadian rhythms in their mRNA expression in synchronized Caco-2 cells (Figure 3). Experimental time series were fitted to equation 12 (cf. Materials and Methods) and a common period of 26 h 50 (SD 63 min) was found (Table 1). Oscillations were damped for all genes except *BMAL1* and *CES2*. Some variability was encountered between the four experiments which explained the weak amplitude of *CES2* rhythm (Text S1). Despite the circadian variations of *TOP1* mRNA expression no consistent rhythm was found in its nucleic protein level (data not shown).

**Table 1:** Parameter values of circadian mRNA expressions in synchronized Caco-2 cells.

	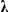 ( 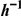 )	*P* (a.u.)	*S* (a.u.)	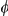 (h, min)	*R* (a.u.)
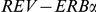	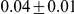	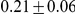	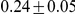	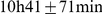	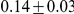
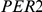	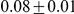	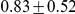	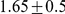	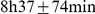	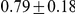
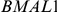	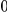	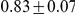	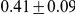	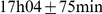	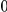
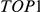	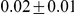	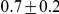	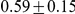	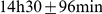	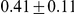
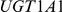	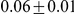	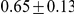	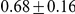	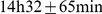	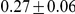
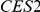	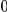	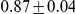	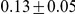	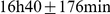	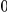
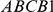	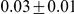	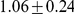	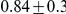	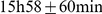	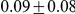
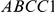	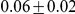	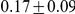	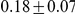	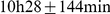	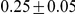
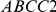	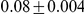	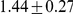	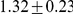	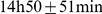	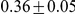
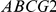	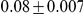	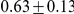	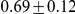	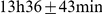	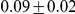

Values correspond to 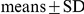. They were estimated by fitting equation 12 to experimental data of Figure 3 by a bootstrap approach (Text S1), a.u. =  arbitrary units.

**Figure 4:**
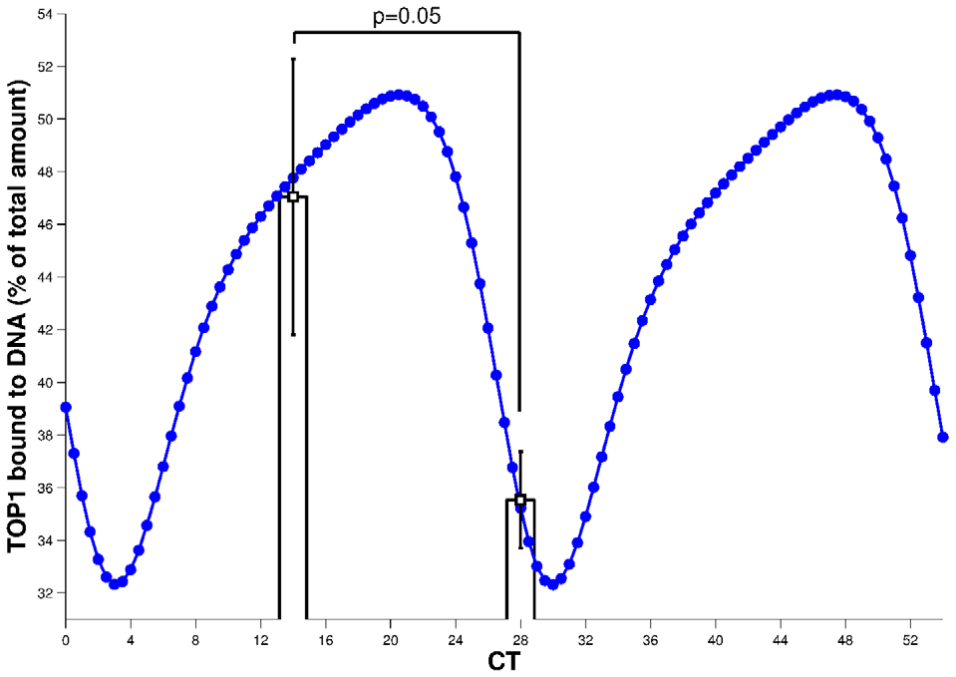
Circadian rhythm of CPT11-induced TOP1 complexes on DNA in synchronized Caco-2 cells. Experimental results are averages of four independent experiments (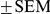) after exposure to CPT11 at CT14 and CT28. The dotted line is the circadian variation of TOP1 complexes (sum of variables *DNATOP1*, *Compl* and *Icompl*) computed using the PK-PD mathematical model fitted to all experimental data (see Results).

DNA-bound TOP1 protein amount was equal to 47% (SEM 5.2%) of TOP1 total protein quantity after exposure to CPT11 at CT14 as compared to 35.5% (SEM 1.8%) after exposure at CT28 (p = 0.05; Figure 4).

### CPT11 molecular PK-PD mathematical model

The CPT11 PK-PD model describes the molecular pathways occurring within a population of quiescent Caco-2 cells exposed to the drug. The modeled biological system consists in one million of Caco-2 cells attached to the bottom of a Petri dish and covered with extracellular medium. 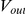 stands for the volume of extracellular medium and is set to 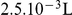. 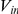 represents the total intracellular volume equal to the experimentally-determined volume of a single cell multiplied by the number of cells. It is equal to 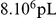. Mathematical variables represent concentrations in the extracellular compartment or intracellular concentrations averaged on the cell population.

The mathematical model of CPT11 molecular PK-PD computes the cytotoxicity induced in Caco-2 cells by any given exposure schedule. CPT11 activity is assessed by the amount of irreversible DNA/TOP1/SN38 complexes, chosen as the output variable because of its experimentally-proven correlation with CPT11 cytotoxicity [26], [27].

Molecular pathways are modeled according to information from literature and experimental results obtained in Caco-2 cells. Briefly, CPT11, SN38 and SN38G cellular uptakes are assumed to be passive as these molecules are mainly under their carboxylate form at the pH of experiments (pH 7.8). Cellular uptake is modeled as a diffusion across a membrane, the contact surface between cells and extracellular medium being proportional to the number of cells [31]. CPT11 and SN38 efflux are mediated respectively by 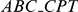 (mainly standing for the sum of activities of ABCB1, ABCC1, ABCC2) and 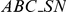 (for ABCC1, ABCC2, ABCG2). Efflux follows Michaelis-Menten kinetics [32], [33]. Diffusion from inside to outside of the cells is neglected. CPT11 is bioactivated into SN38 through CES representing the sum of all carboxylesterases activity. This pathway is also modeled by Michaelis-Menten kinetics [34]–[36].
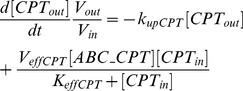
(1)

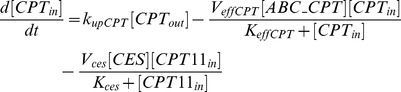
(2)


SN38 is expected to be deactivated into SN38G in Caco-2 cells as UGT1A1 was expressed (Figure 3). This reaction is modeled by Michaelis-Menten kinetics [19], [34], [37]. The mathematical variable UGT stands for the sum of UGT1As enzymatic activities.
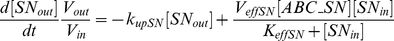
(3)

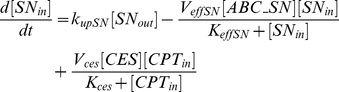
(4)

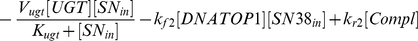
(5)


As SN38G is inactive, its extra- and intracellular concentrations do not exert any influence on the output of the model which is the drug activity. Nevertheless, those quantities can be computed using the following equations in which transport parameters of SN38G were assumed to be equal to the ones of SN38, for the sake of simplicity:
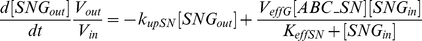
(6)

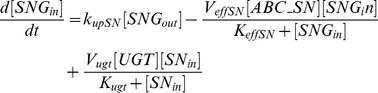
(7)


CPT11 ability to bind to TOP1 is neglected so that SN38 is the only molecule able to stabilize DNA/TOP1 complexes into DNA/TOP1/SN38 ones (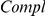). Those ternary complexes are able to spontaneously dissociate or can be converted into irreversible complexes (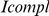) after collision with transcription or replication mechanisms. The 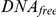 variable represents the number of available binding sites for TOP1 on the DNA (Text S1). Those entry sites are assumed to occur every 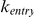 pairs of bases. DNA total quantity is considered as constant because Caco-2 cells are quiescent. Therefore the amount of available binding sites can be expressed as: 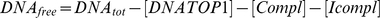.
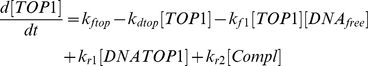
(8)

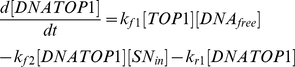
(9)

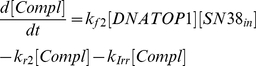
(10)

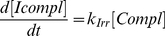
(11)


We consider that the first period of oscillations was artificially perturbed by the serum shock and start the simulation at the second one from which steady oscillations are expected in mRNA and protein amounts. Protein intracellular concentrations are modeled as the result of a constant degradation (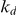) and a circadian translation of period 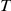, the intrinsic one, mean value M, amplitude A and phase 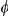. 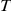 is set to the period determined using the mRNA quantifications shown in Figure 3. 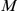 and 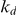 are set to 1 such that the mean value of the protein concentration is equal to 1. Those parameter values result in a delay between mRNA and protein amounts approximately equal to 1h30, a duration accounting for protein synthesis in cultured cells. For each gene, 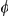 is set to the phase estimated from the corresponding mRNA measurement (Table 1). Thus 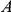 is the only parameter to be estimated for each protein. It is searched in the interval [0, 1]. This range does not imply any assumption about the protein circadian variation as the amplitude A can be equal to zero which corresponds to an absence of rhythm. Of note, the modeled protein concentrations only account for functional proteins and can thus be interpreted as protein activities.
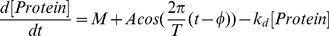
(12)


Although *TOP1* mRNA expression displayed robust circadian variations, its nucleic protein level was constant in Caco-2 cells. Therefore no circadian control is assumed on TOP1 protein amount (see equation 7). As experimental transcriptional results showed circadian rhythms for *UGT1A1*, *CES2* and four ABC transporters and as no information is available on corresponding activities, we assume possible circadian variations for glucuronidation (UGT), bioactivation (CES) and efflux of CPT11 (ABC_CPT) and SN38 (ABC_SN). Equation 12 is used to model them.

Parameter estimation was performed by a bootstrap approach using experimental results in Caco-2 cells and information from the literature. The first step of the estimation consisted in determining correct search intervals for each parameter, which we did by using unpublished data on Caco-2 cells together with existing information from literature (Text S1). For some parameters (e.g. 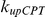), the search interval was very narrow and this first step constituted the essential effort of estimation. The second step consisted in fitting the model to the biological results on CPT11 and SN38 pharmacokinetics (Figure 2) and CPT11 chrono-pharmacodynamics (Figure 4). This was performed by a bootstrap approach in which 50 datasets were generated from the original data. Then the model was fitted to each of these 50 datasets by a least-square approach in which the minimization task was performed by the CMAES algorithm [38]. We thus got 50 parameter sets from which we computed the mean and standard deviation of each parameter (Text S1).

When fitting the model to the experimental results of Figure 2, verapamil exposure was assumed to exert an influence only on the parameter 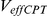 which stands for the activity of CPT11 efflux transporters. Therefore all the parameters were assumed to be the same in presence or absence of verapamil except 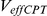 which becomes 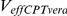 (Table 2).

**Table 2:** Parameter values of the CPT11 molecular PK-PD model.

Reaction	Symbol	Value
CPT11 uptake speed	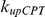	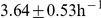
CPT11 efflux	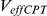	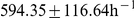
	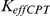	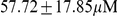
CPT11 efflux in presence of verapamil	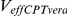	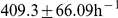
CPT11 activation	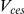	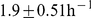
	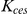	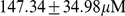
SN38 uptake	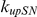	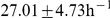
SN38 efflux	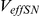	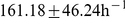
	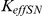	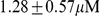
SN38 glucuronidation	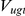	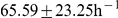
	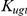	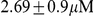
DNATOP1 complex formation	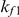	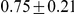 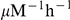
DNATOP1 complex dissociation	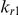	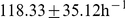
DNA/TOP1/SN38 complex formation	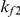	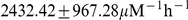
DNA/TOP1/SN38 complex dissociation	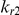	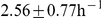
Irreversible complex formation	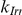	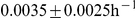
Entry sites on DNA for TOP1 binding	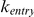	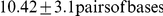
TOP1 protein formation	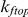	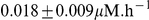
Amplitude of CPT11 bioactivation circadian rhythm	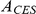	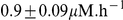
Amplitude of CPT11 efflux circadian rhythm	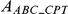	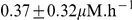
Amplitude of SN38 efflux circadian rhythm	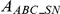	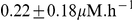
Amplitude of SN38 glucuronidation circadian rhythm	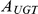	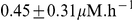

Values correspond to 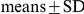. They were estimated by fitting experimental data on Caco-2 cells by a bootstrap approach (Results and Text S1).

 The parameter estimation provided a value for CES circadian amplitude which was by far larger than that of all other proteins. The amplitude of CES was actually greater than that of UGT, 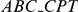, and 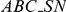 in all the 50 parameter sets computed by the bootstrap approach (Table 2). The standard deviations of 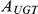, 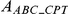 and 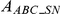 were in the same range as the parameter values which suggested that experimental data could still be fitted by the model even if those circadian amplitudes were close to zero (Table 2). This prefigured a weak influence of those protein circadian rhythms on CPT11 chronotoxicity in Caco-2 cells.

### Theoretical optimization of CPT11 exposure

For therapeutics optimization, well-synchronized Caco-2 cells were considered as healthy cells whereas non-synchronized cells represented cancer cells since circadian organization is often disturbed in tumor tissues [8], [39]. Mathematically, healthy and cancer cells were simulated using the same mathematical model of CPT11 PK-PD. Parameter values were the same except for circadian amplitudes A which were set to zero for tumor cells.

The considered exposure schemes consisted in an administration of CPT11 at a particular initial concentration, starting at a particular CT, over a duration ranging from 1 to 27 h. The cumulative dose is here defined as the initial concentration of CPT11 multiplied by the exposure duration. We chose such schemes because they are easily reproducible in cell culture. CPT11 remained in the blood of cancer patients over approximately 24 h at a concentration ranging from 0 to 20 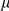
M [8]. Therefore, we investigated cumulative doses ranging from 0 to 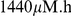 which corresponded to an exposure to 0 to 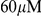 of CPT11 over 24 h. The optimization procedures which follow consisted in determining the optimal values of the cumulative dose, the CT at which the exposure started and its duration.

#### Maximizing efficacy without toxicity constraint

**Figure 5:**
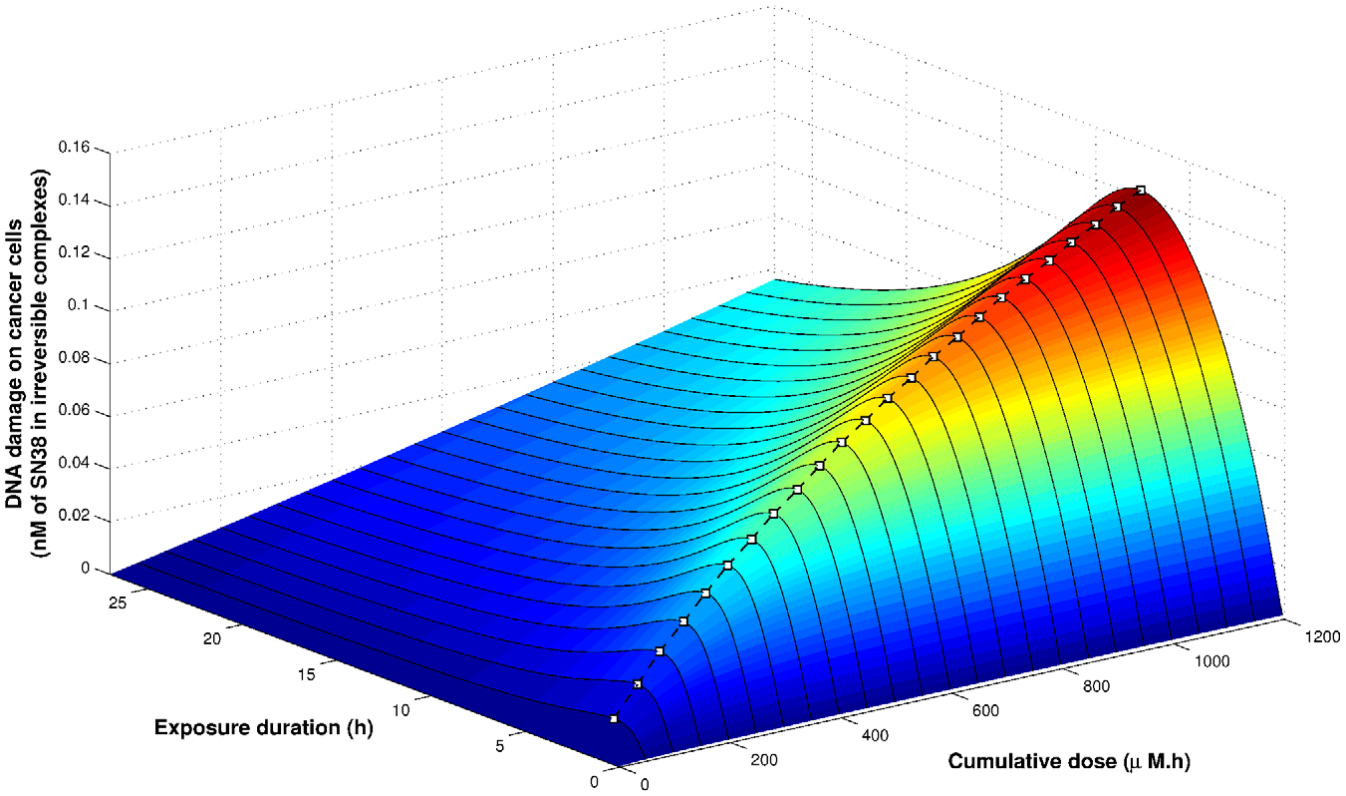
Mathematical optimization of CPT11 exposure in non-synchronized Caco-2 cells. Efficacy in non-synchronized cells with respect to the cumulative dose and the exposure duration. White dots are the most efficient duration for each cumulative dose.

We firstly only considered cancer cells and aimed at maximizing efficacy without taking into account CPT11 toxicity on the healthy cell population. As tumor cells are not synchronized, the CT of exposure has no influence. We therefore looked for optimal values of the cumulative dose and the exposure duration which induced the largest DNA damage. Mathematically, this damage was assessed by the value of the variable 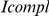 at the end of the exposure. It was computed for a cumulative dose ranging from 0 to 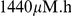 and an exposure duration between 1 and 27 h (Figure 5). For all the considered cumulative doses, optimal durations ranged from 1h50 to 7h40 and increased with the dose. As expected, the maximum efficacy for a given cumulative dose increased with the cumulative dose.

#### Minimizing toxicity without efficacy constraint

**Figure 6:**
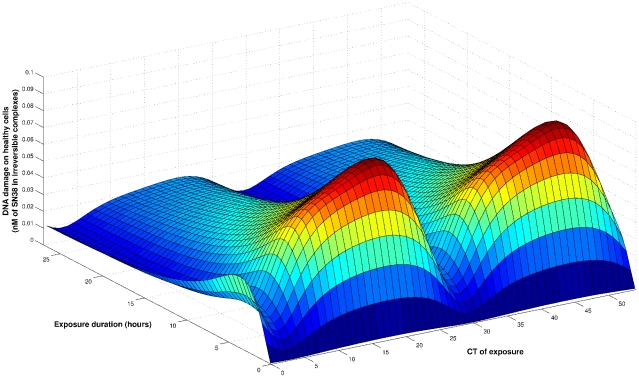
Mathematical optimization of CPT11 exposure in synchronized Caco-2 cells. Toxicity in synchronized cells with respect to exposure duration and CT of beginning of exposure. The cumulative dose was set to 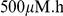.

We then only considered healthy cells and aimed at minimizing toxicity without any constraint of minimum efficacy. Toxicity was here assessed by the value of DNA damage 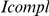 in the normal cell population, at the end of the exposure. The cumulative dose of CPT11, the CT at which the exposure started and its duration were the three parameters considered here. To study the influence of the CT and the exposure duration, we set the cumulative dose to 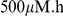 as an example, the following features remaining true for all cumulative doses ranging from 0 to 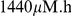. Toxicity was computed for an exposure duration between 1 and 27 h, and a CT of exposure ranging from CT0 to CT54, which corresponded to two periods (Figure 6).

For any exposure duration, the best tolerated scheme was obtained by starting CPT11 administration between CT2 and CT3. This time interval corresponded to 1 to 2 h before the nadir of CES protein amount (Figure 6, 7 A). The most toxic scheme was achieved when CPT11 was administered at CT21 over the exposure duration which also induced the largest DNA damage in cancer cells (4 h for the dose of 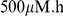). This most toxic duration displayed the largest circadian amplitude in terms of DNA damage induced in healthy cells. CT21 did not correspond exactly to the peak of CES protein amount but was rather the optimal balance between high CES protein amount and low UGT, 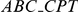 and 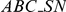 ones.

**Figure 7:**
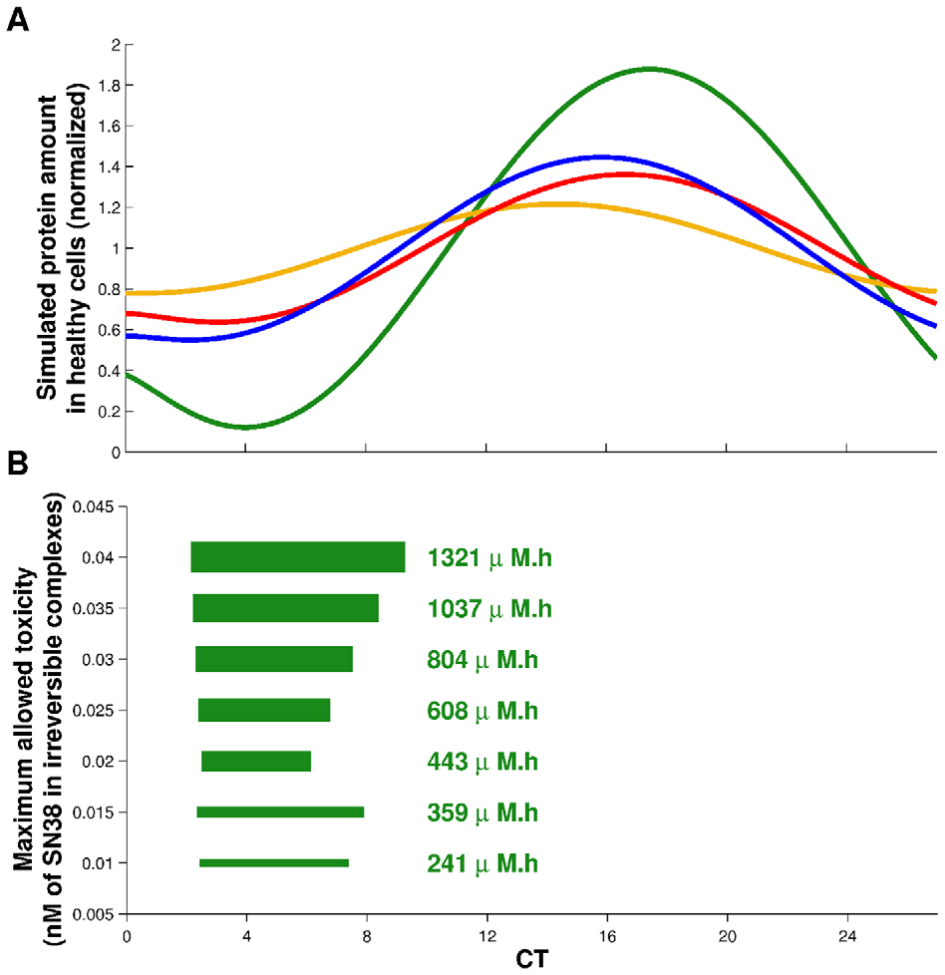
Maximizing CPT11 efficacy under a constrainst of tolerability. **A**: Simulated circadian rhythm of CES (green), UGT (blue), 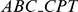 (red) and 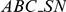 (orange) protein amounts in healthy cells. **B**: Optimal exposure schemes following the strategy of maximizing efficacy under a constraint of maximal allowed toxicity. Optimal schemes consisted in administering the optimal cumulative dose (written in green) over 3h40 to 7h10, starting between 1h30 and 1h50 before the nadir of CES protein amount. Schemes were not centered on the nadir but rather extended after it when UGT, 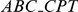 and 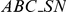 amounts were higher and protected more efficiently healthy cells.

#### Maximizing efficacy under a constraint of minimal toxicity

Finally, in the perspective of control theory [40], we adopted the therapeutics strategy of maximizing DNA damage on cancer cells under the constraint of DNA damage on healthy cells not exceeding a tolerability threshold. In a clinical point of view, this threshold represents the maximum toxicity that the patient can handle and may vary according to gender, genetic background or previous treatments. Healthy and cancer cells were numerically exposed to the same drug schedule mimicking the *in vivo* situation in which healthy and tumor tissues are exposed to the same blood concentrations.

The optimization procedures consisted in determining the optimal values of the cumulative dose, the CT at which the exposure started and its duration. Concerning the numerical algorithm, a cost function was minimized to determine optimal schemes for each value of the tolerability threshold. This function was the sum of two terms: the first one was the opposite of DNA damage 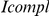 in cancer cells, the second one was the toxicity constraint which took the form of an *If* statement equal to 0 if 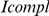 in healthy cells was below the toxicity threshold and 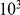 otherwise. The weight on the constraint was intentionally very high in order to unconditionally maintain the toxicity under the threshold. The algorithm CMAES was used to minimized the cost function. It was preferred to Matlab function *fmincon* as it was able to handle the large discontinuities of the cost function due to the constraint. We investigated tolerability thresholds ranging from 0.01 to 0.04 nM of SN38 bound to DNA which corresponded to the studied range of cumulative doses.

Computed optimal cumulative doses of CPT11 ranged from 241 to 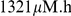 and increased with the tolerability threshold. For any dose, the optimal scheme consisted in administering CPT11 over 3h40 to 7h10 starting between CT2h10 and CT2h30 which corresponded to 1h30 to 1h50 before the nadir of CES protein amount (Figure 7). The optimal schemes were not centered on the nadir of 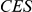 rhythm but rather extended after it, when 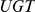, 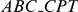 and 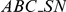 amounts were higher and therefore protected more efficiently healthy cells. For any maximum allowed toxicity, the optimal duration did not exceed 7h10 highlighting the need of short exposure durations to optimally exploit the temporal difference between healthy and cancer cells. Regarding efficacy, those optimal schemes induced twice more DNA damage in cancer cells than in healthy ones.

## Discussion

We gave evidence for a circadian organization in Caco-2 cells resulting in rhythms in CPT11 PD. A mathematical model of CPT11 molecular PK-PD was designed, fitted to experimental results and used for therapeutics optimization. It concluded that any dose of CPT11 should be optimally administered over a duration of 3h15 to 7h10, starting between 1h30 and 1h50 before the nadir of CPT11 bioactivation rhythm.

A clinical interpretation of the optimal therapeutics strategies presented in this *in vitro* study can be obtained by rescaling the period from 27 h, that of Caco-2 cells, to 24 h. It thus suggests to administer CPT11 such that it remains over an active concentration in the patient blood during 3h30 to 6h30, starting between 1h20 and 1h40 before the nadir of the patient's circadian rhythm of CESs. In cancer patients, a dumbbell delivery scheme over 6h resulted in CPT11 circulating during approximately 12 h [8]. The present study thus suggests to reduce the duration of CPT11 infusion which may enhance efficacy. This might also lead to a decrease in the total administered dose in order to achieve acceptable tolerability.

SN38 was detected in Caco-2 cells which is in agreement with the fact that CES2 was expressed. Its efflux was not influenced by verapamil suggesting a weak activity of the inhibitor on transporters responsible for SN38 efflux. Indeed, ABCG2 for which SN38 is a good substrate, is poorly inhibited by verapamil [41]. Another possibility is that SN38 efflux was not mediated by active transporters, the highly lipophilic molecules of SN38 diffusing passively across the cell membrane.

CPT11 is converted by cytochromes P450 3A4/3A5 into two metabolites: APC and NPC [16]. Those pathways were not taken into account as they are reported to be inactive in most Caco-2 cell lines [42]. Nevertheless verapamil inhibits cytochrome P450 3A [43] and the drastic increase of CPT11 intracellular concentration in the presence of verapamil could be explained by the decrease in CPT11 conversion into APC and NPC.

Only 0.1% of CPT11 dose was bioactivated into SN38 in Caco-2 cells (Figure 2). Our data suggest that the remaining 99.9% exerted weak cytotoxicity as described in the literature [17]. Indeed verapamil exposure of Caco-2 cells resulted in a 10-fold increase in the intracellular concentration of CPT11 without any change in that of SN38. In contrast with those large pharmacokinetics differences, the average percentage of surviving cells after exposure to verapamil and CPT11 was only reduced by 11.6% relative to that measured when cells were exposed to CPT11 alone. In the light of those experimental results, it seemed justified to neglect CPT11 activity in the current mathematical model.

The protein degradation (i.e. 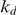) was assumed to be constant as no biological data were available regarding its circadian variations for each considered enzyme. However, this assumption must be tempered since circadian changes could modulate the phosphorylation processes occurring upstream of protein degradation [44].

The mathematical model predicted that CPT11 bioactivation through CES was the main determinant of CPT11 PD circadian rhythm. Even though the circadian amplitude of *CES2* mRNA expression was the lowest among all measured genes, its protein amount and activity could be highly rhythmic. Indeed CES1 protein level displayed robust circadian variations in the colon mucosa of two mouse strains despite low amplitude of the circadian variations of its mRNA level [2]. Nevertheless, the 1h30-delay between mRNA and protein expression assumed in the mathematical model might be underestimated [45] and UGT1A1 or ABC transporters may have been determinant for CPT11 PD rhythm. However this hypothesis was not favored as the shifts between their mRNA and protein expressions must have exceeded ten hours to be relevant.

Here, a realistic interval of values was determined for each parameter. Thus our mathematical model constitutes a reasonable tool to explore therapeutics optimization in the perspective of clinical applications. However, more biological information is needed to determine precise values of all parameters in the Caco-2 cell line and therefore be able to predict its quantitative response to CPT11.

CPT11 activity was assessed by the amount of irreversible CPT11/TOP1/DNA complexes. Those complexes trigger DNA repair and possibly lead to apoptosis [46]. Many repair enzymes as well as proteins involved in the apoptotic machinery such as p53, the pro-apoptotic BAX or the anti-apoptotic BCL-2 display circadian rhythms which may influence CPT11 chronotoxicity [2], [47]–[49]. Therefore DNA repair and apoptosis enzymes should both be studied in Caco-2 cells and included in an extended version of the mathematical model.

In the current model we differentiated cancer cells from healthy ones by their lack of circadian entrainment. The proliferation rate constitutes another important difference which was not considered here as we studied quiescent cells. Our mathematical model has been supplemented with a circadian entrained cell cycle model in order to optimize CPT11 exposure in proliferating cells [50].

This study on cell populations is being further integrated into a whole-body physiologically-based PK-PD model which aims at investigating molecular differences between experimentally-determined chronotoxicity mouse classes and designing optimal administration schemes for each of them [2]. This prefigures the part of mathematical modeling in the determination of patient classes characterized by molecular biomarkers according to which the circadian delivery would be tailored. In the case of colorectal cancer, patients often undergo surgery after which tumor and healthy cells can be collected and analyzed at the molecular level [36], [51]. The whole body mathematical model could then be fitted to the patient molecular profile and provide a tailored chronomodulated administration scheme.

## Materials and Methods

### Cell culture

The Caco-2 cell line was obtained from the American Type Culture Collection (Rockville, MD). Cells were grown in Dulbecco's modified Eagle's: Ham F12 medium (1∶1) supplemented with penicillin (100 U/L), streptomycin (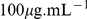), glutamine (2 mM) (Fischer Scientific, Paris, France) and 10% of Fetal Bovine Serum (FBS) (Dutscher, Paris, France). They were maintained in a humidified atmosphere containing 5% 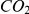 at 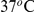. Experiments were performed four days after confluence. CPT11 was purchased from Pfizer (Paris, France). For transport inhibition studies, non-synchronized cells were pre-incubated with verapamil, a non specific inhibitor of ABC transporters (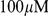 for 24 h; Sigma Aldrich, Paris, France) [52]. Cells were then exposed to CPT11 (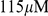) during 48 h. Cell synchronization was performed by a serum shock which consisted in a 2-hour exposure to serum rich medium (DMEM:F12 containing 50% FBS). The beginning of the serum shock defined Circadian Time 0 (CT 0).

### RNA quantification

Circadian gene expressions were estimated using quantitative Real-Time Polymerase Chain Reaction (qRT-PCR). Cells were scraped at different CTs in guanosine isothiocyanate and frozen at 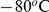 until RNA extraction performed as described in [53]. Reverse transcription was achieved with Superscript II RT (Invitrogen, Paris, France). Quantitative PCRs were performed with LightCycler 480 using LightCycler 480 SYBR Green I master kit (Roche, Meylan, France). Relative quantification of target RNA using 36B4 as reference was performed with Relquant software (Roche, Meylan, France).

### CPT11 lactone-carboxylate equilibrium

CPT11 mother solution being at pH 4.4, CPT11 underwent a transformation from its lactone to carboxylate form when it was added to the culture medium at pH 7.8. In order to assess the duration of this reaction, we built a mathematical model and fitted it to data from [54] (Text S1). In our experimental conditions the model predicted that the lactone/carboxylate equilibrium was reached within 3 h. The culture medium containing CPT11 was therefore prepared more than 3 h before incubation with cells.

### Drug concentration measurement

CPT11 and SN38 extra- and intracellular concentrations were measured by a method of High Performance Liquid Chromatography (HPLC) adapted from [55]. After cell exposure, extracellular medium was sampled. The cell layers were rinsed with Phosphate Buffer Saline (PBS), recovered by scraping and pelleted in 1 mL of PBS. Cells were then centrifuged. Cell pellets were resuspended in 100 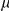
L of water supplemented with 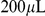 of methanol/acetonitrile (50/50 v/v) containing 1% HCl. Cells were centrifuged again and the supernatant containing drugs was sampled. 70 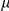
L was diluted by adding 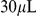 of water and spiked onto the column. Extracellular samples were diluted ten times, pretreated with acetonitrile 1% trifluoroacetic acid and centrifuged before injection. Separation was carried out on a C-18 reversed-phase column (Interchim, Montlucon, France) with a mobile phase consisting of a mixture of water, acidified acetonitrile (0.005% of trifluoroacetic acid) and methanol (50∶38∶12, v/v/v). This mobile phase was delivered isocratically at a flow rate of 0.6 mL/min with a P680 pump (Dionex Corporation, Sunnyvale, CA). Fluorometric detection was carried out with excitation and emission wavelengths set at 380 and 532 nm respectively, using the RF-2000 detector (Dionex Corporation). Peaks were quantified by reference to a standard calibration curve obtained by spiking known amounts of drugs (CPT11 (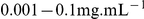) and SN38 (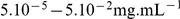)), using WinNonLin Pro software (Pharsight Corporation, Mountain View, CA). The values obtained from HPLC measurement were normalized to one million cells (Text S1).

### TOP1 activity

The Topo I Link Kit (TopoGen, Port Orange, FL) was used to quantify the amount of TOP1 linked on DNA [56]. The cells were lysed with 3 mL lysis buffer. Cell lysates were loaded at the top of a cesium chloride gradient and centrifuged at 100,000 g for 16 h at 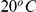. Fractions of 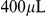 were removed from the top of the gradient, and an aliquot of each fraction (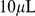) was diluted (1/5) and quantified at 260 nm with spectrophotometer (Eppendorf, Paris, France). In parallel, another aliquot of each fraction (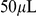) was diluted with an equal volume of 25 mM sodium phosphate buffer and loaded onto a nitrocellulose membrane (Perbio, Paris, France) using a slot-blot device. TOP1 was revealed in the slots with the immunoblotting technique described in the TopoGen kit using ProteinA-peroxidase (1/5000, Sigma Aldrich, Paris, France) to replace secondary antibody. A signal was seen in two different groups of slots: those not containing DNA (free TOP1, top of the gradient) and those containing DNA (DNA/TOP1 complexes, bottom of the gradient). Chemoluminescence signals were detected with Las4000 camera and quantified with ImageJ software. For circadian assessment of TOP1 complexes after CPT11 exposure cells were exposed during 30 min at 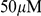 at indicated CT. The software SPSS (IBM, Somers, NY) was used for the Anova test (univariate general linear model).

### Gene expression mathematical model

Circadian gene expressions in synchronized Caco-2 cells were modeled as a damped cosine:
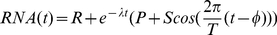
(13)


The damping factor 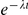 was both applied to the cosine to represent cell desynchronization over time and to 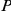 to model the initial overexpression of genes due to the serum shock. Both dampening exponents were assumed to be equal for the sake of simplicity. 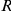 represented the mRNA value before the serum shock towards which the model converges in long time. All genes were assumed to oscillate with the same period which was the cell population intrinsic one. This common period 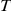 and gene parameters 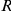, 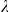, 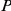, 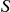, and 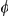 were estimated simultaneously for all genes using a bootstrap approach to fit experimental results of Figure 3 (Text S1).

### Minimization algorithms

Matlab function *fmincon* and the *CMAES* algorithm [38] were used for minimization tasks in parameter estimations and optimization procedures.

## Supporting Information

Text S1 Supporting information concerning Materials and Methods and parameter estimation.
(PDF)
