# Supplementary material for: A Combined Experimental and Mathematical Approach for Molecular-based Optimization of Irinotecan Circadian Delivery
Source: PLoS Comput Biol. 2011 Sep 8;7(9):e1002143. doi: 10.1371/journal.pcbi.1002143 (PMC3169519; doi:10.1371/journal.pcbi.1002143)
Supplement: Text S1 — Supporting information concerning Materials and Methods and parameter estimation. (PDF) [file pcbi.1002143.s001.pdf]

Text S1: Supporting Information

1 Materials and Methods

1.1 mRNA quantification

Circadian mRNA expressions were quantified by real-time polymerase chain reaction (RT-PCR). Primers used for amplification of clock genes ($REV-ERB\alpha$, *PER2*, *BMAL1*) and reference (36B4) were described previously ([S1]). The others were ABCB1 sense (CCACAGAGGGGATGGTCAGT), antisense (TAGGCATTGGCTTCCTTGAC), ABCC1 sense (ACCATCCACGACCCTAATCC) and antisense (GACTTGTTCCGACGTGTCCT), ABCC2 sense (ACCTAGGCACATGGCTCCTG) and antisense (CAGGATCTGGAATCCGTAGG), ABCG2 sense (CCCAGTGTCACAAGGAAACA) and antisense (ACGAAGATTTGCCTCCACCT), UGT1A1 sense (TCCCAGGAATTTGAAGCCTA) and antisense (GTGATAAAGGCACGGGTCAT), TOP1 sense (CCAAGCATAGCAACAGTGAAC) and antisense (GAGGCTCGAACCTTTTCCTC), CES2 sense (GTTAACGCTGCTGATGTTGC) and antisense (GGTGGCCTGATGTTCTTGAG). Gene accession numbers were for ABCB1 NM_000927.3, for ABCC1 NM_004996.3, for ABCC2 NM_000392.3, for ABCG2 NM_004827.2, for UGT1A1 NM_000463.2, for TOP1 NM_003286.2 and for CES2 NM_003869.5. Hybridization temperature for all the primers was 60^oC.

1.2 The lactone-carboxylate model

A mathematical model was built to study the equilibrium between the lactone and the carboxylate forms of CPT11 with respect to the pH of the solution. The temperature was assumed to be constant and equal to 37°C. We considered that reactions occurred in a closed system containing a buffer solution which kept the pH constant. Two inverse chemical reactions occurred:

$$\text{At high pH: } CPT_{lactone} + HO^- \xrightarrow{k_c} CPT_{carboxylate}$$
$$\text{At low pH: } CPT_{carboxylate} + H_3O^+ \xrightarrow{k_l} CPT_{lactone} + H_2O$$

The speed of those reactions clearly depends on concentrations of ions $[H_3O^+]$ and $[HO^-]$.

To model this problem we defined two variables: CPT_l and CPT_c respectively CPT11 lactone and carboxylate concentrations expressed in μM. As the system was closed, the total quantity of CPT-11 was conserved over time: $CPT_c + CPT_l = CPT_{tot}$. Applying the law of mass action and using this conservation law gave the following equation for CPT_l kinetics:

$$\frac{dCPT_l}{dt} = -(k_l[H_3O^+] + k_c[HO^-])CPT_l + k_l[H_3O^+]CPT_{tot}$$

We then introduced the pH as $[H_3O^+] = 10^{-pH}$ and $[HO^-] = 10^{pH-14}$. This led to:

$$\frac{dCPT_l}{dt} = -(k_l 10^{-pH} + k_c 10^{pH-14})CPT_l + k_l 10^{-pH} CPT_{tot}$$

As the pH remained constant because of the buffer solution we obtained a simple ODE that was solved analytically:

$$CPT_l(t) = (CPT_l(0) - \tfrac{B}{A})e^{-At} + \tfrac{B}{A}$$

with $A = k_l 10^{-pH} + k_c 10^{pH-14}$ and $B = k_l 10^{-pH} CPT_{tot}$

At equilibrium the following holds:

$$CPT_l^* = \tfrac{B}{A} = \tfrac{k_l 10^{-pH}}{k_l 10^{-pH} + k_c 10^{pH-14}} CPT_{tot}$$

We used experimental data in Table I and Table II of [54] to estimate k_c and k_l by a least square approach using the CMAES algorithm for minimization. Estimated parameter values were: $k_l = 19300\text{h}^{-1}$ and $k_c = 8.54.10^{-6}\text{h}^{-1}$ (Figure S1, panel 1 and 2). We then validated our model by comparing it to the set of data contained in Figure 6 in [54] (Figure S1, panel 3).

The mother solution of CPT11 is at pH 4.4. In our experimental conditions, cells were cultured in an extracellular medium at pH 7.8. We investigated the needed time to reach the lactone/carboxylate equilibrium after the mother solution was added to the culture medium. Thus we searched for t such that: $CPT_l(t) - CPT_l^* \leq eps$ where eps was a tolerance parameter. At pH=4.4, all CPT-11 molecules were under their lactone form: $[CPT_l](0) = [CPT_{total}] = 115\mu M$. We ended with the analytical formula:

$$t \geq \tfrac{1}{A}(ln(CPT_l(0) - \tfrac{B}{A}) - ln(eps)).$$

Replacing numerical values in this formula and setting eps to 10^{-3} gave : $t \geq 2.9$. Therefore CPT11 was added to the medium at least 3 hours before using it for cell exposure so that the lactone/carboxylate equilibrium was reached.

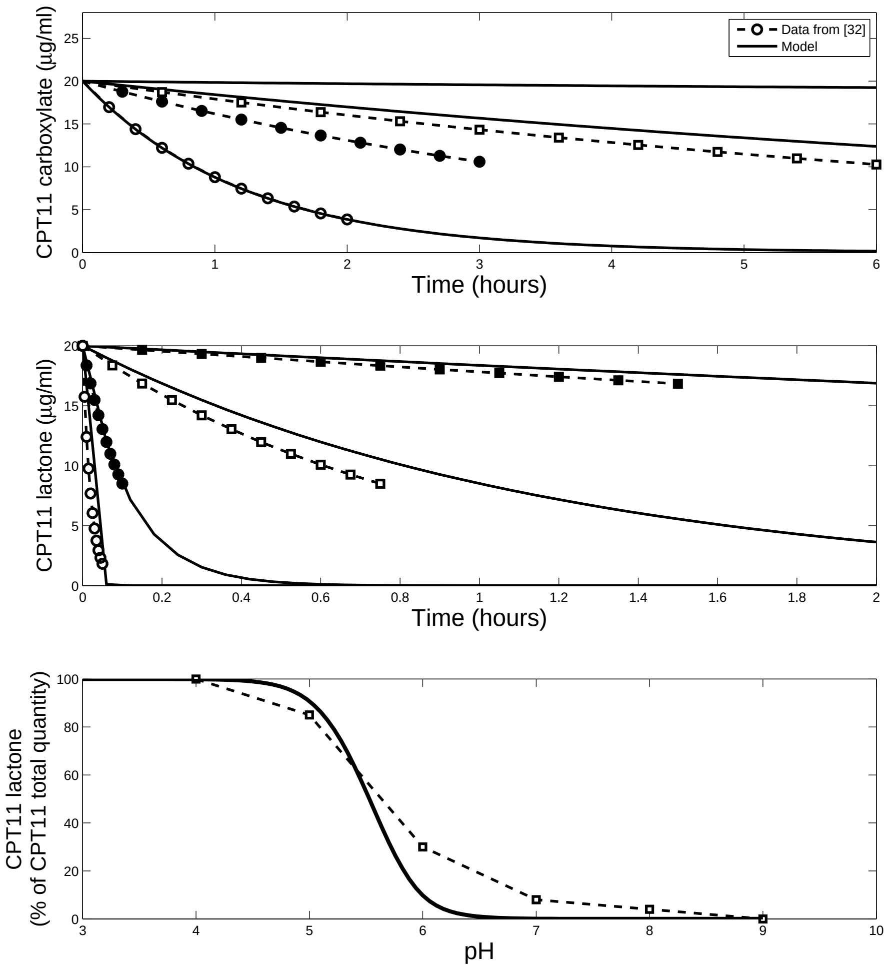

Figure S1: **The lactone/carboxylate model.** Panel 1: CPT11 carboxylate transformation into CPT11 lactone at pH 4 (◯), 5 (•) and 6 (□). Panel 2: CPT11 lactone transformation into CPT11 carboxylate at pH 6 (■), 7 (□), 8 (•) and 9 (◯). Panel 3: Percentage of CPT11 lactone over CPT11 total quantity at equilibrium with respect to pH. See [54] for details about experimental results.

1.3 Normalization of HPLC measurements

CPT11 and SN38 extra- and intracellular concentrations were measured by HPLC. The direct results obtained from HPLC depended on the number of cells present in the Petri Dish and were therefore normalized as if all Petri dishes contained one million of cells. Control Petri dishes after 2, 12, 24 and 42 h of exposure were trypsinized and their cell number counted using the Malassez cell. Then we interpolated to obtain the number of cells for the other exposure durations.

HPLC measurements were normalized as follows. Let denote n the quantity of moles of drug and N_c the measured number of cells in a Petri dish. $()_{meas}$ stood for the measured value from HPLC, $()_{1M}$ for the normalized value to one million cells. For intracellular concentration, the following holds: $(n_{in})_{1M} = \frac{(n_{in})_{meas}}{N_c}$. Therefore we get:

$$(C_{in})_{1M} = \frac{(C_{in})_{meas}}{N_c}.$$

For extracellular concentration normalization, we used the conservation law on the drug total quantity to compute $(n_{out})_{1M}$:

$$\begin{aligned}(n_{out})_{1M} &= n_{tot} - \frac{(n_{in})_{meas}}{N_c} \\ (n_{out})_{1M} &= n_{tot} - \frac{1}{N_c}(n_{tot} - (n_{out})_{meas})\end{aligned}$$

Finally we got:

$$(C_{out})_{1M} = \frac{N_c - 1}{N_c} C_{tot} - \frac{1}{N_c}(C_{out})_{meas}$$

1.4 Parameter estimation for circadian gene expressions in synchronized Caco-2 cells

The circadian expression of ten genes were measured in Caco-2 cells, namely three clock genes *REV-ERBα*, *PER2*, and *BMAL1*; three metabolism and target genes *TOP1*, *UGT1A1*, *CES2*; and four ABC transporters *ABCB1*, *ABCC1*, *ABCC2* and *ABCG2*. We assumed that those genes oscillated with the same period T. Experimental data of mRNA expression were fitted to equation (1) (cf. Materials and Methods). In addition to the common period T, four parameters have to be estimated for each gene: R, λ, P and S. The 41 parameters were estimated simultaneously using a Bootstrap approach. Briefly, data were first normalized so that each mRNA expression had an average value equal to 0 and a standard deviation equal to 1. Then five hundreds datasets were generated by assuming that mRNA values at each time point followed a Gaussian law of average and standard deviation the ones of the corresponding data time point. For each generated dataset, parameters were estimated using a least square approach. The minimization of the cost function was performed using the Matlab function *fmincon* in which interval boundaries were specified for all parameters. Only few constraints of intervals were active: for *PER2*, *ABCC1*, *ABCC2*, *ABCG2* the upper bound on λ was active ($\lambda < 0.085$); for *TOP1* and *ABCB1*, the lower bound on R was active ($R > -1.5$), for *UGT1A1* the upper bound on λ was active ($\lambda < 0.07$). Finally the mean value and standard deviation of the hundred parameter sets were computed and rescaled to obtain non-normalized values.

2 Circadian rhythms of mRNA amounts : variability between experiments

The mRNA amounts of *CES2*, *TOP1*, *UGT* and $REV-ERB\alpha$ were quantified in four independent experiments (Figure S2). *CES2* circadian rhythm was consistent in experiment 1, 2 and 4 with an amplitude of 15%, 29%, and 28% of their mean value respectively. On the contrary, no obvious circadian pattern of CES2 expression was noticed in experiment 3. The overshoot in $REV-ERB\alpha$ expression during the first cycle in experiments 1, 2 and 4 was not observed in experiment 3.

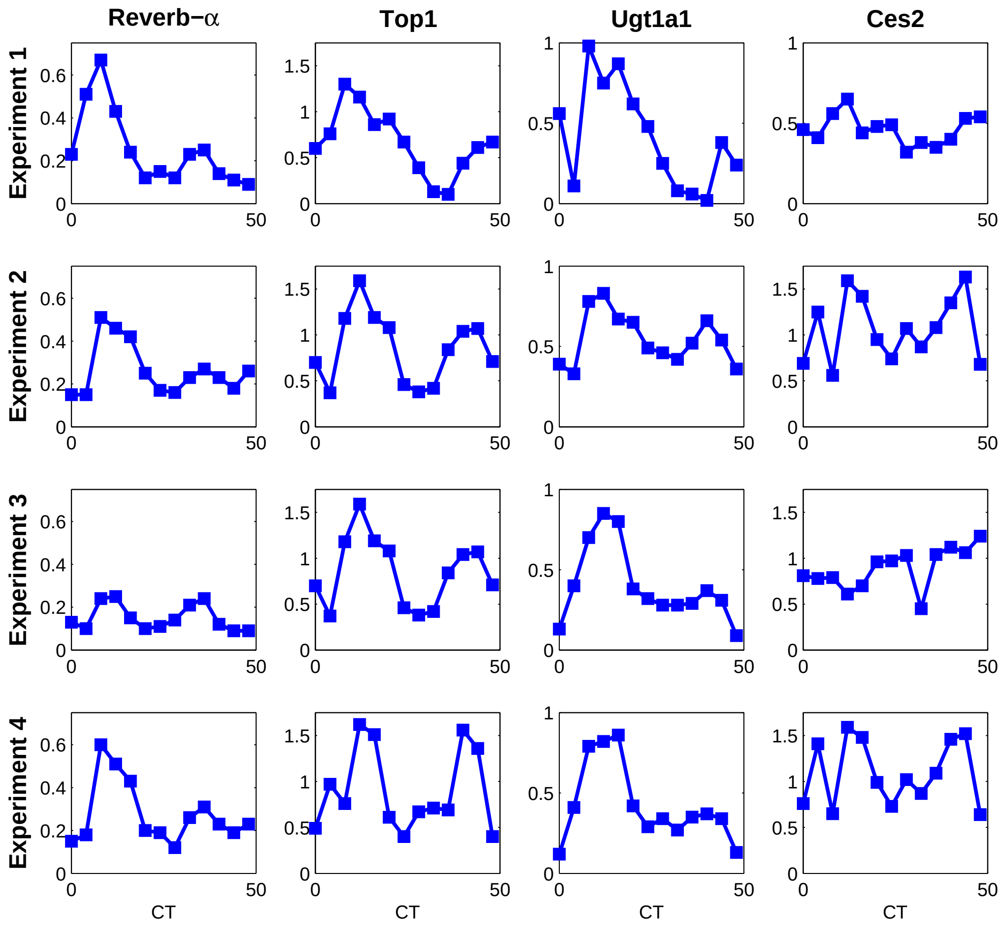

Figure S2: **Circadian rhythm of mRNA quantity of $REV-ERB\alpha$, *TOP1*, *UGT1A1* and *CES2* in four independent experiments**

3 Parameter estimation for the CPT11 molecular PK-PD model

This section aims at giving more details about how the parameters of the CPT11 molecular PK-PD model were estimated. The first step consisted in determining reasonable search intervals for each parameter either based on literature or on unpublished data on our Caco-2 cell line. Then the second step used a bootstrap approach to fit the model to experimental data shown in Figures 2 and 4.

3.1 Determining a search interval for each parameter

Search intervals for parameters were determined as follows:

- CPT11 Uptake: CPT11 uptake was assumed to occur passively at the rate k_{upCPT}. It was studied in Caco-2 cell culture by measuring CPT11 intracellular concentration after a 10-minute exposure at concentrations from 10 to 100μM (data not shown). We assumed that in the first ten minutes CPT11 efflux could be neglected as the drug had only time to enter the cell ([20]). This allowed the computation of an approximation of the uptake parameter $k_{upCPT} = 5.9\text{h}^{-1}$. A deviation of $\pm$50% was allowed in parameter estimation.

- SN38 Uptake: SN38 uptake was assumed to occur passively at the rate k_{upSN}. As no measurement of SN38 uptake was available in our Caco-2 cells, we used experimental data from [20] to determine SN38 uptake parameter and found: $k_{upSN} = 15.3\text{h}^{-1}$. A deviation of $\pm$100% was allowed in parameter estimation.

- CPT11 Efflux: CPT11 efflux followed Michaelis-Menten kinetics with parameters V_{effCPT} and K_{effCPT}. We used data from Figure 2 and unpublished data on CPT11 accumulation in Caco-2 cells at concentrations 10μM, 40μM to compute a guess for the efflux parameters. Indeed once the parameter for CPT11 uptake was approximately known, it was possible to deduce CPT11 efflux parameters from equation (2) by approximating the derivative of $[CPT11_{out}]$ by a forward Euler scheme. We found: $V_{effCPT}[ABC\_CPT] = 1170\mu\text{M.h}^{-1}$ and $K_{effCPT} = 71\mu\text{M}$. A deviation of $\pm$50% was allowed in parameter estimation.

- CPT11 bioactivation into SN38 by CESs:

  the transformation of CPT11 into SN38 enhanced by CESs followed Michaelis-Menten kinetics with parameters V_{ces} and K_{ces}. As no experimental information was available concerning CPT11 bioactivation kinetics in Caco-2 cells we used data from literature. In [35], they found K_{ces}=53 μM and V_{ces}=0.714 $\mu\text{M.h}^{-1}$ with [CES]=2.03 μM. In [34], they found $K_{ces} = 53\mu\text{M}$ and $V_{ces} = 1.018\mu\text{M.h}^{-1}$ with $[CES] = 1.42\mu\text{M}$. Therefore we searched for $V_{ces}[CES]$ in the interval [0.5, 5] and for K_{ces} in [20 150].

- SN38 efflux: SN38 efflux followed Michaelis-Menten kinetics with parameters V_{effSN} and K_{effSN}. Those parameters were directly inferred from experimental data of Figure 2 and 4.

- SN38 deactivation into SN38G by UGT1As: the glucuronidation of SN38 into SN38G catalyzed by enzymes UGT1As followed Michaelis-Menten kinetics with parameters

V_{ugt} and K_{ugt}. No measurement of SN38G concentration was available in Caco-2 cells. Using the experimental results of [34] the following values were computed: $V_{ugt}[UGT] = 27\mu$M.h^{-1} and $K_{ugt} = 2.13\mu$M. Therefore we searched for $V_{ugt}[UGT]$ in the interval [0, 90] and for K_{ugt} in [0, 5].

- Formation and dissociation of DNA/TOP1 reversible complexes: we first determined an approximation of TOP1 total concentration by assuming that all SN38 molecules present in the intracellular medium in Figure 2 were trapped in complexes with TOP1. SN38 intracellular concentration was equal to 0.01μM in average and data from Figure 3 suggested that around 40% of TOP1 total quantity was trapped in complexes with the drug. Therefore TOP1 total concentration could be estimated to 0.025μM and was searched within the interval [0 0.1]. In the absence of drug, around one third of TOP1 was linked to the DNA in Caco-2 cells (data not shown) which led to the following initial values for the formation (k_{f1}) and dissociation (k_{d1}) rates of DNA/TOP1 complexes: $k_{f1} = 2$, $k_{d1} = 300$. A deviation of ±100% was allowed for those two parameters.

- Formation and dissociation of DNA/TOP1/SN38 reversible complexes: the association (k_{f2}) and dissociation (k_{r2}) rates of SN38 with DNA/TOP1 complexes were directly inferred from experimental data of Figure 2 and 4.

- Formation of irreversible complexes: the irreversible complexes come from collision of reversible complexes with replication or transcription mechanisms which occur at the rate k_{Irr}. We assumed that this rate was independent of time meaning that the amount and speed of mechanisms going along the DNA was constant. This assumption was realistic in quiescent cells but would be untrue in proliferating cells in which the number of replication forks strongly increases during the S-phase. No prior information was available concerning the parameter k_{Irr} which was directly fitted to experimental data of Figures 2 and 4.

- DNA total quantity: entry sites where TOP1 can bind to the DNA were assumed to occur every k_{entry} pairs of bases. Here we defined an entry site as k_{entry} pairs of bases. When a molecule of TOP1 associated with an entry site, it was consumed and then became a *Compl* molecule. As a cell contains approximately $3.2.10^9$ pairs of base, the number of moles of entry sites was $\frac{3.2.10^9}{N_A k_{entry}}$ where N_A is the Avogadro number. Thus the concentration of free entry sites in a cell with a volume equal to $V_{cell} = 8.10^{-12}$L is approximately: $DNA_{tot} = \frac{3.2.10^9}{N_A k_{entry} V_{cell}} M$. k_{entry} was assumed to be between 1 and 100. Therefore DNA_{tot} was searched within the interval [5 500].

- Protein amount circadian amplitudes: circadian amplitudes for CES, UGT, ABC_CPT and ABC_SN were searched in the interval [0, 1] so that the protein formation term in equation (12) remained positive.

3.2 A bootstrap approach for the second step of parameter estimation

The second step of our parameter estimation consisted in the simultaneous evaluation of the 21 parameters which were searched in the intervals previously determined. The mathematical model was fitted to experimental data on CPT11 and SN38 pharmacokinetics in the presence or absence of verapamil (Figure 2) and CPT11 chrono-pharmacodynamics measured at CT14 and CT28 (Figure 4). Parameters were estimated using a Bootstrap approach. Briefly, fifty datasets were generated from the original one, by assuming that

concentrations at each time point followed a Gaussian law of mean and standard deviation the ones measured at the corresponding data time point. For each generated dataset, parameters were estimated using a least square approach in which the minimization task was performed by the CMAES function. This algorithm was preferred to Matlab function *fmincon* because it handled better local minima. Thus we got 50 parameter sets from which we derived the mean value and standard deviation for each parameter (Table 2).

In order to start the 50 least-square estimations with initial values close to the optimal ones, we firstly determined a preliminary fit. We fitted averaged experimental values of Figures 2 and 4 by a least square approach which was iterated for different initial values of parameters. We thus computed the parameter set which fitted the best the average experimental data and used it as initial values for the bootstrap approach.

References

S1. Teboul M, Barrat-Petit MA, Li XM, Claustrat B, Formento JL, et al. (2005) Atypical patterns of circadian clock gene expression in human peripheral blood mononuclear cells. J Mol Med 83: 693-9.
